# Germany’s digital health reforms in the COVID-19 era: lessons and opportunities for other countries

**DOI:** 10.1038/s41746-020-0306-7

**Published:** 2020-07-10

**Authors:** Sara Gerke, Ariel D. Stern, Timo Minssen

**Affiliations:** 1 Project on Precision Medicine, Artificial Intelligence, and the Law; Petrie-Flom Center for Health Law Policy, Biotechnology, and Bioethics at Harvard Law School, Harvard University, Cambridge, MA USA; 2 Technology and Operations Management Unit, Harvard Business School, and Harvard-MIT Center for Regulatory Science, Boston, MA USA; 3 Centre for Advanced Studies in Biomedical Innovation Law (CeBIL), Karen Blixens Plads 16, 2300 Copenhagen, DK USA

**Keywords:** Health policy, Health services

## Abstract

Reimbursement is a key challenge for many new digital health solutions, whose importance and value have been highlighted and expanded by the current COVID-19 pandemic. Germany’s new *Digital Healthcare Act* (Digitale–Versorgung–Gesetz or DVG) entitles all individuals covered by statutory health insurance to reimbursement for certain digital health applications (i.e., insurers will pay for their use). Since Germany, like the United States (US), is a multi-payer health care system, the new Act provides a particularly interesting case study for US policymakers. We first provide an overview of the new German DVG and outline the landscape for reimbursement of digital health solutions in the US, including recent changes to policies governing telehealth during the COVID-19 pandemic. We then discuss challenges and unanswered questions raised by the DVG, ranging from the limited scope of the Act to privacy issues. Lastly, we highlight early lessons and opportunities for other countries.

A recent survey of 284 health care, life science, and digital health professionals in the United States (US) revealed that 42% of respondents felt they were “likely” or “somewhat likely” to partner or contract with an AI company over the next year^1^. However, a significant share of respondents also believed that digital health partnerships face unique obstacles with regard to key issues such as pricing and reimbursement (26%) and data privacy and security (19%)^1^. Consequently, they were reluctant to collaborate with digital health companies for a variety of reasons. Of note was the fact that 60% of respondents believed that “strongly entrenched business and reimbursement models make it difficult to bring digital health products to market,” highlighting reimbursement as a key challenge for many new digital health solutions^1^. The ongoing COVID-19 pandemic has emphasized and expanded the importance of digital health technologies, ranging from a rapid transition to telehealth services^2^ to the development of contact tracing and warning apps^3^.

Despite many differences, Germany, like the US, has a multi-payer health care system with over 100 independent insurers, making it an especially interesting case study for American policymakers^4^. On November 7, 2019, the German parliament (Bundestag) adopted the *Digital Healthcare Act* (Digitale-Versorgung-Gesetz or DVG)^5^, which was subsequently approved by the Federal Council (Bundesrat) and signed into law by the German President. In addition to promoting the use of telehealth and ensuring better usability of health data for research purposes, the new law entitles all individuals covered by statutory health insurance to benefits for certain digital health applications (i.e., insurers will pay for their use)^6^.

While many consider the DVG to be a breakthrough in incentivizing and advancing patients’ diagnosis, management, and treatment with digital health tools, the new law has also attracted criticism and faces a number of challenges moving forward. In this article, we explain the relevant changes encompassed in the new German DVG and provide an overview of reimbursement of digital health solutions in the US, including changes to telehealth delivery and reimbursement during the COVID-19 pandemic. We then discuss challenges and unanswered questions raised by the DVG and highlight early lessons and opportunities for other countries.

## The new German Digital Healthcare Act

The German statutory health insurance system is one of the largest in the world^7^. Approximately 90% of the population (i.e., roughly 75 million people) in Germany are covered by statutory, state-funded health insurance, while the remaining 10% are privately insured^7,8^. By primarily making amendments to the *Social Security Code V* (Sozialgesetzbuch V—SGB V)^9^, the DVG entitles those insured by one of Germany’s independent statutory health insurance providers to coverage benefits for certain digital health applications. This means that insurers will be required to pay for qualifying applications, making such digital health solutions broadly accessible.

**Fig. 1:**
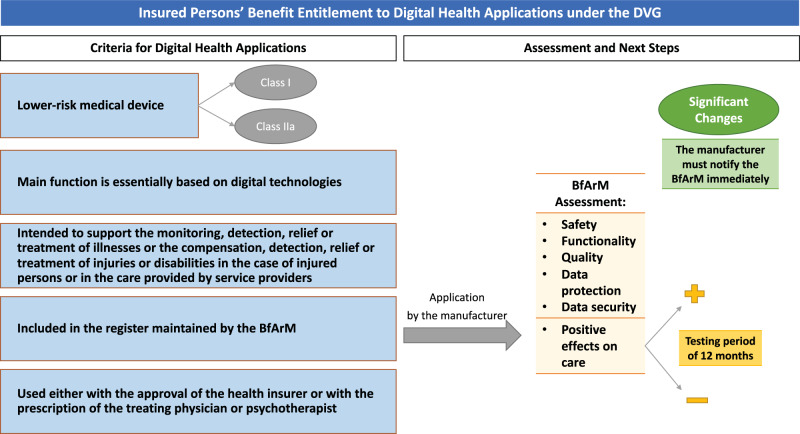
Overview of the Criteria and Assessment Process for Digital Health Applications under the German Digital Healthcare Act (DVG). Criteria for digital health applications (left column) and assessment by the BfArM to be included in the register and next steps (right column).

In general, insured persons are entitled to coverage benefits for digital health applications if such applications meet the following criteria:

1. They are lower-risk medical devices;
2. Their main function is essentially based on digital technologies;
3. They are intended to support the monitoring, detection, relief or treatment of illnesses or the compensation, detection, relief or treatment of injuries or disabilities in the case of injured persons or in care provided by service providers;
4. They have been included in a newly established official register for digital health applications maintained by the German Federal Institute for Drugs and Medical Devices (Bundesinstitut für Arzneimittel und Medizinprodukte—BfArM); and
5. They are used either with the approval of the health insurer or with the prescription of the treating physician or psychotherapist (SGB V, § 33a(1)) (Fig. 1).

Lower-risk medical devices as defined by the first requirement are those classified as class I or class IIa medical devices under the European Union (EU) Medical Device Regulation 2017/745 (MDR)^10^ or the EU Medical Device Directive 93/42/EEC (MDD)^11^ (Box 1)^12^.

To meet the fourth requirement, each digital health application must be included in a newly established register maintained by the BfArM. At the request of the manufacturer, the BfArM will assess whether the manufacturer has provided proof that their digital health application fulfills the following requirements: safety, functionality, quality of the medical device, data protection, state-of-the-art data security, and positive effects on care (SGB V, § (2)) (Fig. 1). The BfArM will make a decision regarding the manufacturer’s request within 3 months of receiving complete application documents (SGB V, § 139e(3)).

If the manufacturer satisfies all requirements except the last one (i.e., positive effects on care—“positive Versorgungseffekte”), the digital health application can be included in the register for a preliminary (testing) period of 12 months (SGB V, § 139e(4)) (Fig. 1), during which time the manufacturer must provide evidence of its effects on care. The explanatory memorandum clarifies that such effects constitute one of two things: (1) a medical benefit (i.e., a therapeutic improvement by positively influencing patient-relevant endpoints such as quality of life), or (2) procedural and structural improvements to health care—e.g., the promotion of patient information and patient sovereignty, better coordination of care processes, etc^6^. The memorandum also clarifies that the medical benefit can be demonstrated via expert opinions, case reports, application observations, studies, or other valid findings^6^. If the manufacturer does not manage to provide sufficient evidence within the first year, the BfArM can extend the deadline for a further 12 months if, based on the preliminary information submitted, there is a high likelihood of later verification (SGB V, § 139e(4)).

Most recently, on April 9, 2020, the Digital Health Applications Ordinance (Digitale Gesundheitsanwendungen-Verordnung—DiGAV)^13^ entered into force. The DiGAV regulates the procedure and requirements for review of reimbursement eligibility of digital health applications by statutory insurers^13^. In particular, the ordinance lays out details for inclusion of digital health applications in the register, including means for demonstration of evidence of positive effects on care. The BfArM has meanwhile also published guidelines for manufacturers that interpret the DiGAV and provide supplementary details on the procedure for inclusion in the register (SGB V, § 139e(8))^12^.

Once a digital health application is included in the register, the National Association of Statutory Health Insurance Funds (“GKV-Spitzenverband”) begins the process of negotiating a standardized reimbursement price (“negotiated price”) with the manufacturer (SGB V, § 134(1)). This price then applies to all statutory health insurers 12 months after the entry of a digital health application into the register (SGB V, § 134(1)). During the first year, the manufacturer’s list price applies (SGB V, § 134(5)). In the case that the manufacturer and the National Association of Statutory Health Insurance Funds cannot agree on a negotiated price within one year, an arbitration board will set the reimbursement price within three months (SGB V, § 134(2)). This process has many similarities to that of reimbursement for new pharmaceuticals by German statutory health insurers, in which negotiated prices only apply after the first year of the product being placed on the market following a formal benefit assessment (SGB V, § 130b)^6^.

In the event that the manufacturer makes “significant changes” to a digital health application that is included in the register, it must notify the BfArM immediately (SGB V, § 139e(6) no. 1) (Fig. 1). The BfArM then decides within 3 months after the notification whether the application should be removed from the register or whether the register should be adjusted to reflect the updated product’s features (SGB V, § 139e(6)). If significant changes are not reported, a penalty of up to 100,000 Euros can be imposed (SGB V, § 139e(6)).

The DVG also aims to accelerate the adoption and use of telehealth. In particular, under the DVG, patient can more easily take advantage of video consultations. During such a consultation, a patient can be informed about circumstances that are essential for consent to a medical measure, including its nature, scope, implementation, expected risks, and consequences (SGB V, § 291g(4); BGB^14^, § 630e)^6^. Previously, this was only possible following a consultation in the physician’s office before a video consultation^6^. Moreover, physicians in Germany can now advertise video consultations as long as proper treatment and advice, based on the recognized state of medical knowledge, are possible (HWG^15^, § 9)^6^.

The use and value of telehealth tools such as video consultations have grown during the COVID-19 pandemic. Due to the spread of the virus, the National Association of Statutory Health Insurance Physicians (“Kassenärztliche Bundesvereinigung”) and the National Association of Statutory Health Insurance Funds lifted the limitations on the number of cases and amount of services that can be provided via video consultations^16^. Previously, only every fifth patient could be treated by a doctor or psychotherapist via video consultation and such consultations were limited to 20% of total services provided^16^. While doctors can now use video consultation flexibly in all therapeutically relevant cases, psychotherapists are limited to video consultation for certain services and may only practice telehealth following an in-person consultation involving an initial diagnosis, indication, and the provision of relevant information^16^. For telehealth visits, physicians and psychotherapists must use one of over 30 certified video service providers^17^ and typically have to notify their Association of Statutory Health Insurance Physicians (“Kassenärztliche Vereinigung”) that they are using a certified vendor to be eligible to bill for services^17^.

The DVG also contains provisions to make demographic data from health insurers more usable for research purposes (SGB V, §§ 303a to 303f). In particular, in accordance with the EU General Data Protection Regulation 2016/679 (GDPR), the DVG allows certain beneficiaries such as universities and publicly funded research institutions (e.g., the Max Planck Society) to process certain demographic data from health insurers for specific research purposes, especially for analysis of treatment or care processes or longitudinal analysis over longer periods (SGB V, §§ 303b, 303e(1) and (2))^6^. Such data may include information on patient gender, age, place of residence, vital status, date of death, and billing data like hospital treatment invoices (SGB V, § 303b(1)). If researchers can clearly explain the suitability and necessity of the scope and structure of the requested data, a responsible research data center will transmit the data to them in an anonymized and aggregated format (SGB V, § 303e(3)). Under certain conditions, individual data records can also be provided in pseudonymized form (SGB V, § 303e(4)). Researchers may only use such data for agreed-upon purposes, and the data can typically not be passed on to third parties (SGB V, § 303e(5)). The details of such data sharing research agreements will be further regulated through a new ordinance by the German Federal Ministry of Health (SGB V, § 303a).

Box 1 Medical devices under the new MDR MDR’s date of application:

- The new MDR will repeal, inter alia, the MDD. Due to the COVID-19 pandemic and the accompanying increase in demand for medical devices such as ventilators, the MDR’s date of application was postponed by one year to May 26, 2021^38^.

Definition of the term “medical device”:

- The new MDR defines the term “medical device” broadly as “any instrument, apparatus, appliance, software, implant, reagent, material or other article intended by the manufacturer to be used, alone or in combination, for human beings for one or more of the following specific medical purposes: – diagnosis, prevention, monitoring, prediction, prognosis, treatment or alleviation of disease, – diagnosis, monitoring, treatment, alleviation of, or compensation for, an injury or disability, (…) and which does not achieve its principal intended action by pharmacological, immunological or metabolic means, in or on the human body, but which may be assisted in its function by such means” (MDR, Art. 2(1)).

Classification of medical devices:

- As is the case under the MDD, under the new MDR, medical devices will be classified into four categories based on their risk (ranging from the lowest to highest):

➔Class I, class IIa, class IIb, and class III (MDR, Art. 51(1))^39^.

Software:

- The MDR clarifies that “software, which drives a device or influences the use of a device, shall fall within the same class as the device. If the software is independent of any other device, it shall be classified in its own right” (Rule 3.3 in Chapter II of Annex VIII).
- The MDR also specifies the details of the classification of software. For example, “software intended to monitor physiological processes is classified as class IIa” only in cases where it is not “intended for monitoring of vital physiological parameters, where the nature of variations of those parameters is such that it could result in immediate danger to the patient” (Rule 11 in Chapter III of Annex VIII)^39,40^.

## Reimbursement of digital health solutions in the US

In the US and other Organisation for Economic Co-operation and Development (OECD) countries, there are few formal, large-scale mechanisms for the reimbursement of digital health solutions. However, recent developments, including COVID-19, have led to increased coverage of other digitally provided services, such as the use of “telehealth”—a term that broadly refers to “the delivery of health care, health education, and health information services via remote technologies”^18^.

A key player in the US health care system is the federal Medicare Program, which provides insurance coverage for over 60 million Americans^19^. Since 2019, Medicare Part B (outpatient medical insurance) has provided insurance coverage for some telehealth services such as office visits, psychotherapy, and other consultations, with more telehealth benefits expected for beneficiaries of Medicare Advantage (plans offered by private companies approved by Medicare) in 2020^20^.

During the COVID-19 pandemic, Medicare has meaningfully expanded its telehealth coverage policies. For example, all Medicare beneficiaries can now receive telehealth services provided by clinicians, regardless of whether they are new or established patients and where they are located^21^. The (temporary) ability of clinicians to provide care across state lines represents a dramatic departure from historic US practice regulations. Moreover, health care providers have also been given the option to waive copayments for such services for beneficiaries of Medicare Part B^21^. Further, a wider range of practitioners can now offer telehealth services to patients; these include speech language pathologists, occupational therapists, and physical therapists^21^. Beneficiaries of Medicare Advantage may also receive more telehealth benefits than had previously been the case^20^.

The US market is large, and coverage of specific products and services—beyond mandated essential health benefits^22^ and those that are medically necessary—are determined by individual payers^23^. However, Medicare’s coverage determinations typically play an outsized role in shaping other payers’ coverage strategies and are a precursor to private coverage in several, but not all contexts^24^. As the nation’s largest and most influential payer, it is therefore interesting to explore reimbursement approaches by Medicare and its parent agency, the US Center for Medicare and Medicaid Services (CMS).

Historically, Medicare has reimbursed for health care services on a fee-for-service basis—i.e., “doctors and other health care providers are paid for each service performed”^25^. Such a reimbursement strategy is straightforward from a billing perspective but may fail to incentivize quality of care or value, defined as health outcomes achieved per dollar spent^26^. In a fee-for-service system, digital health solutions face an uphill battle to reimbursement: Few existing billing codes apply to such tools, so manufacturers are left to pursue alternative strategies for getting paid, such as asking clinicians to pay for digital tools^27^.

During the COVID-19 pandemic, the use of telehealth services has skyrocketed^28^. This can be attributed to the multiple temporary changes introduced to telehealth coverage policies described above^29^. In particular, to promote the use of telehealth services, Medicare announced that health care providers can now bill for a telehealth visit at the same rate as if the visit had been in-person^30^. Some private insurers have also introduced payment parity for telehealth^2^. However, changes to telehealth coverage policies will only formally extend through the COVID 19 pandemic, and, as such, new post-COVID-19 models of care delivery and reimbursement will need to be developed that maintain the broad use of telehealth services, where efficient, but avoid overpayment through (typically) shorter telehealth compared to in-person visits^2^.

Beyond telehealth, in the absence of a broad set of standards for digital health reimbursement such as those introduced in Germany through the DVG, even clinically-proven digital health solutions struggle to define a viable reimbursement strategy in the US market, as neither patients nor physicians believe that they should bear the cost of such tools. Moreover, payers, who stand to benefit most from the widespread adoption of effective digital health solutions due to their potential to reduce overall costs, are not operationally equipped to ensure their clinically appropriate adoption and/or ongoing compliant use^31^.

Absent coverage rules and guidelines, other reimbursement strategies have been highlighted as a potential path forward for the use and reimbursement of digital health solutions in the US and other countries. One example of such a reimbursement strategy is the use of bundled payments. In contrast to fee-for-service reimbursement, bundled payments involve a single price for all care required to treat a particular medical condition of a patient^32^. In cases where a “bundle” is used, any tools that have the potential to reduce overall costs of care—e.g., through reduced hospital readmissions—will become attractive for health care providers, even if they increase some input costs. In the past decade, CMS has piloted the use of bundles through the Bundled Payments for Care Improvement Initiative^33^. Eligible settings for bundles include acute episodes of care such as coronary artery bypass graft surgery and major joint replacement as well as a few chronic conditions such as chronic obstructive pulmonary disease and diabetes. All of these conditions are likely to benefit from digital health solutions, with the greatest long-term potential likely to come from tolls that facilitate the management of chronic conditions, which will create value on an ongoing basis.

Both national coverage rules—such as those created by Germany’s DVG—as well as the increased use of value-based reimbursement approaches such as bundled payments can stimulate uptake of digital health solutions. However, combining both new reimbursement approaches and national coverage rules would go even further to stimulate the development and adoption of innovative, beneficial digital health solutions. Although both approaches encourage the use of proven solutions, not all medically beneficial solutions will be promoted via value-based payment models—only the subset of offerings that drive down overall condition-related care costs. National coverage rules, such as those laid out in the DVG go a step further to ensure that other evidence-based digital health solutions will have a clear path to reimbursement in the formal health care delivery system.

## Challenges and unresolved questions

While the new German law has been welcomed by many—and by developers of digital health solutions in particular—it faces a variety of challenges and unresolved questions.

### Limited scope of the DVG

One criticism is the limited scope of the DVG since the Act only applies to lower-risk medical devices, and many potentially valuable digital health applications are therefore not covered by the provisions of the DVG. First, non-medical devices fall a priori outside of the DVG. Second, digital health applications that are categorized as class IIb or class III devices are also not covered by the Act. For example, certain clinical decision support software under the new MDR (“software intended to provide information which is used to take decisions with diagnosis or therapeutic purposes (…) [and] have an impact that may cause (…) death or an irreversible deterioration of a person’s state of health (…) or a serious deterioration of a person’s state of health or a surgical intervention”) will fall outside of the scope of the DVG (Rule 11 in Chapter III of Annex VIII of the MDR). Under the new MDR, software that influences the use of a device or drives a device falls within the same class as the device itself (Rule 3.3 in Chapter II of Annex VIII). Thus, for example, a software app that drives an implantable device like a pacemaker would be classified as a high-risk device and would fall outside of the scope of the DVG’s provisions. Third, the DVG does not apply to those class I- and class IIa-devices that fail to fulfill the other requirements (see above), such as those whose main function is not essentially based on digital technologies.

### Challenges for manufacturers to be included in the register for reimbursable digital health applications

Manufacturers face several hurdles in pursuing inclusion in the newly established register for reimbursable digital health applications maintained by the BfArM. In particular, the satisfaction of the last requirement—i.e., demonstration of positive effects on care—will certainly create challenges. At launch, many manufacturers will likely not be able to demonstrate sufficient evidence of the efficacy of their applications. Although an application can be included in the register for an initial testing period of 12 months, manufacturers need to submit a plausible justification for the likely contribution of their application to improving care i.e., at least the results of a systematic data analysis for the use of the application (DiGAV, § 14)—and a scientific evaluation concept for the demonstration of positive effects on care created by an independent institution (SGB V, § 139e(4)). Further, positive effects on care can only be demonstrated in the course of the testing period by showing medical benefit or patient-relevant procedural and structural improvements to care delivery^13^. The DiGAV and the BfArM guidelines regulate the details of such a demonstration^12,13^. In particular, a comparative study must be carried out (usually in Germany), showing better outcomes with versus without the relevant digital health application (DiGAV, § 10).

### Negotiation of prices

The question of how to calculate and agree upon negotiated prices after the first 12 months of listing in the BfArM register may also pose challenges. Much will depend on negotiations between the National Association of Statutory Health Insurance Funds and the manufacturer of a digital health application; these parties need to agree on a standardized reimbursement price that will apply to all statutory health insurers. The DVG’s requirement to include outcome-related components as a subject of price agreements (SGB V, § 134(1)) will likely raise additional challenges. Moreover, the process is non-transparent since negotiations are held behind closed doors, and minutes and advisory documents are confidential (SGB V, § 134(1)). Thus, it is particularly important for the success of the DVG to ensure the quality and efficacy of the procedural set-up and the usability of tools that support these complex and confidential negotiations. Furthermore, although the manufacturer retains the right to charge a higher selling price than the negotiated price (the difference would need to be paid by the insured individual)^6^, manufacturers will likely stick to the negotiated prices in order to maximize demand for their products.

### Risk of penalty payments

Manufacturers can face a penalty of up to 100,000 Euros if they fail to notify the BfArM immediately about significant changes to their digital health application (SGB V, § 139e(6)). Although the BfArM can only set this penalty payment after: (1) having given the manufacturer a deadline for notification—usually not more than four weeks—and, (2) the manufacturer has also missed this deadline (SGB V, § 139e(6)), the financial consequences for manufacturers, especially small and medium-sized enterprises, could be fatal. The DiGAV (§ 18) and the BfArM guidelines contain some examples of what is meant by “significant changes”^12,13^ for instance, a change in the location of the data storage^12^. The BfArM also provides a case report form on its website to help manufacturers assess whether a change to a digital health application is “significant”^12^. In addition, the BfArM offers advice on any questions the manufacturer may have^12^.

### Privacy issues

Some patient advocates have raised the criticism that the DVG does not enable patients to opt-out of sharing their demographic data collected by health insurers for research purposes^34^, with some data privacy activists filing an unsuccessful petition in mid-November 2019 asking the German president to not sign the DVG into law^35^. There is also the risk of reidentification: in a world of artificial intelligence and big data, it is possible that requested data may be reidentified through algorithms when combined with other available data from a beneficiary^36^. When processing requested data under the DVG, the beneficiary has to make sure that no connection is made to service providers or people (SGB V, § 303e(5)). However, the Act also acknowledges that such a connection may inadvertently be established, and when it does, it must be reported to the research data center (SGB V, § 303e(5)). For example, a university could potentially re-identify an employee by accidentally combining requested data for research purposes under the DVG with information easily available from medical certificates regarding sick days, leading to the revelation of sensitive information about an employee’s health status. The DVG therefore also contains safeguards to reduce the risk of reidentification. The responsible research data center must assess such risk in relation to the data requested by the beneficiary and take appropriate measures to minimize it while adequately preserving the intended scientific benefit (SGB V, § 303d(1) no. 5).

### Lessons and opportunities for other countries and conclusions

Among OECD countries, Germany’s DVG represents a first-of-its-kind opportunity for large-scale reimbursement of evidence-based digital health applications. Although Germany is one of the world’s biggest health care markets with annual spending of around 374 billion EUR (approximately 417 billion USD), it has historically had one of the lowest levels of digitization among developed countries^34^. The new DVG and its reimbursement standards for digital health applications thus represent an important step in not only modernizing the German health care delivery system but also improving the quality of care for patients and being better prepared for future pandemics.

Yet some challenges and unresolved questions remain, ranging from the limited scope of the DVG to data privacy to hurdles for manufacturers to be included in the newly established register for reimbursable digital health applications maintained by the BfArM. These remaining issues may temper digital health application manufacturers’ enthusiasm as they consider entering the German market. For new digital health applications to be fully accepted by patients, payers, and providers, it will be imperative that outstanding procedural issues are addressed thoughtfully and transparently.

Worldwide, there are over 318,000 mobile health apps available, and around 200 apps are being added to app stores each day^37^. To ensure patient and consumer safety, it is important that health apps are assessed based on criteria such as safety, functionality, quality, data protection, data security, and positive effects on care. We believe that a risk-based approach can serve as a useful starting point for policymakers in the development of standardized, evidence-based assessment processes and legal requirements for digital health solutions. However, policymakers will also need to make sure that they do not overregulate the sector in such a way that it will make it overly burdensome for manufacturers to meet the requirements for qualifying their products for reimbursement, thus preventing or slowing innovation.

As other countries and health systems look to implement coverage policies and assessment processes for digital health solutions, further developments and resolution of questions about the DVG will certainly provide valuable insights. From the perspective of manufacturers, it is clear that well-articulated evidence standards and straightforward assessment processes will help to stimulate the entry of new products. In particular, the BfArM guidelines provide additional procedural clarity and will hopefully help to pave the way for more innovation and entry of digital health manufacturers in Germany. To promote the use of digital health solutions beyond the COVID-19 pandemic, there may also be the option to introduce additional incentives, such as reduced insurance fees. As other countries’ health insurance systems grapple with questions of how and when to reimburse for digital health solutions, policymakers should carefully monitor how Germany’s DVG is implemented in practice and use the German experience to draw lessons and opportunities from the process.

## Data Availability

This article does not make direct use of any data.
